# Slit1 Protein Regulates SVZ-Derived Precursor Mobilization in the Adult Demyelinated CNS

**DOI:** 10.3389/fncel.2020.00168

**Published:** 2020-06-26

**Authors:** C. Deboux, G. Spigoni, C. Caillava, B. Garcia-Diaz, A. Ypsilanti, N. Sarrazin, C. Bachelin, A. Chédotal, A. Baron-Van Evercooren

**Affiliations:** ^1^Institut du Cerveau et de la Moelle épinière-Groupe Hospitalier Pitié-Salpêtrière, INSERM U1127, CNRS, UMR 7225, Sorbonne Université, UM75, Paris, France; ^2^Institut de la Vision, Sorbonne Université, INSERM, CNRS, Paris, France

**Keywords:** Slit1, neural precursor, oligodendrocytes, recruitment, migration, myelin

## Abstract

Slit1 is a secreted axon guidance molecule, also involved in adult neurogenesis. In physiological conditions, Slit1 loss promotes ectopic dispersal of SVZ-derived neural precursors (SVZ-NPCs) into periventricular structures such as the corpus callosum. Demyelination of the corpus callosum triggers SVZ-NPC migration to ectopic locations and their recruitment by the lesion, suggesting a possible role for Slit1 in SVZ-NPCs ectopic dispersal regulation in pathological conditions. Here, we have investigated the function of Slit1 protein in the recruitment of SVZ-NPCs after CNS demyelination. We find that the dynamics of oligodendrogenesis and temporal profile of developmental myelination in *Slit1*^–/–^ mice are similar to *Slit1*^+/−^ controls. SVZ micro-dissection and RT-PCR from wild-type mice, show that Slits and Robos are physiologically regulated at the transcriptional level in response to corpus callosum demyelination suggesting their role in the process of SVZ-NPC ectopic migration in demyelinating conditions. Moreover, we find that the number of SVZ-NPCs recruited by the lesion increases in *Sli1^–/–^* mice compared to *Slit1*^+/−^ mice, leading to higher numbers of Olig2^+^ cells within the lesion. Time-lapse video-microscopy of immuno-purified NPCs shows that Slit1-deficient cells migrate faster and make more frequent directional changes than control NPCs, supporting a cell-autonomous mechanism of action of Slit1 in NPC migration. In conclusion, while Slit1 does not affect the normal developmental process of oligodendrogenesis and myelination, it regulates adult SVZ-NPC ectopic migration in response to demyelination, and consequently oligodendrocyte renewal within the lesion.

## Introduction

The discovery of on-going neurogenesis throughout life in the majority of mammals (Hinds, 1968; Altman, 1969) as well as the identification of neural stem/precursor cells (NPCs) in the adult rodent (Reynolds and Weiss, 1992), non-human primate (Kornack and Rakic, 2001; Pencea et al., 2001) and human CNS (Kirschenbaum et al., 1994) opens new perspectives for self-repair of brain damage (reviewed in Picard-Riera et al., 2004; Nait-Oumesmar et al., 2008; Okano and Sawamoto, 2008; Kaneko and Sawamoto, 2009; Spigoni et al., 2014). Multipotent and self-renewable precursors are located in the hilus of the hippocampus and the subventricular zone (SVZ) of the lateral ventricles of the forebrain, as well as in the spinal cord ependyma (Morshead et al., 1994; Weiss et al., 1996; Ernst et al., 2014). *In vitro*, the SVZ-NPCs, self-expand in response to epidermal growth factor (EGF) and fibroblast growth factor (FGF), and differentiate into neurons, astrocytes and oligodendrocytes upon growth factor retrieval (Reynolds and Weiss, 1992; Lois and Alvarez-Buylla, 1993). *In vivo*, they retain the capacity to divide and migrate in chain through the rostral migratory stream (RMS) to the olfactory bulb (OB) where they become peri-glomerular and granular neurons (Luskin, 1993; Lois and Alvarez-Buylla, 1994). They are repelled from the SVZ by the choroïd plexus and CSF flow, which triggers their migration toward the OB where they disperse and differentiate into olfactory neurons (Hu, 1999). Within the RMS, SVZ-NPCs follow blood vessels and are surrounded by an astrocytic channel, which guides them from the SVZ to the OB (Snapyan et al., 2009; Whitman et al., 2009; Kaneko et al., 2010). In both focal and multifocal demyelinating models, SVZ neuroblasts (type A) and transit amplifying cells (type C cells) proliferate and are mobilized from their normal pathway of migration to be recruited by the lesion where they generate new oligodendrocyte progenitors (OPCs) to participate with parenchymal OPCs, in myelin repair (Nait-Oumesmar et al., 1999; Decker et al., 2002a, b; Picard-Riera et al., 2002; Menn et al., 2006; Aguirre et al., 2007; Jablonska et al., 2010; Tepavcevic et al., 2011; Xing et al., 2014; Brousse et al., 2015). Demyelination induced ectopic migration is preceded by SVZ proliferation and ependymal modifications suggesting a role for ependymal cells in SVZ stem cell signaling in inflammatory-demyelinating conditions (Pourabdolhossein et al., 2017). Unlike in rodents, in multiple sclerosis, NPCs accumulate in the SVZ suggesting their reactivation but failure of recruitment by nearby lesions (Nait-Oumesmar et al., 2007; Tepavcevic et al., 2011). While little is known about the molecular machinery regulating ectopic migration of the adult SVZ-NPCs, gaining insights into this process could promote their involvement in brain repair.

During development and adulthood, NPC migration from the SVZ is under the control of a variety of growth factors (reviewed in García-González et al., 2010), extra cellular matrix components, cell adhesion and guidance molecules (reviewed in Spigoni et al., 2014). While some of these molecular cues differ between the developmental and adult stage reflecting the maturation of the RMS glial tube (Peretto et al., 2005), the Slit proteins remain major regulators of the SVZ-NPC directed migration toward the OB. The Slits are secreted proteins discovered for their role in axon guidance and cell migration (Blockus and Chédotal, 2016). Three Slits, Slit1, Slit2, and Slit3, were identified in mammals and are all expressed in the developing and adult CNS (Brose et al., 1999; Nguyen-Ba-Charvet et al., 1999, 2004; Plump et al., 2002). Slits bind to trans-membrane receptors called roundabout (Robo). In mammals, four Robo receptors were identified, but only two of them, Robo1 and Robo2, bind Slits (Brose et al., 1999; Koch et al., 2011; Zelina et al., 2014). Interestingly, SVZ/RMS NPCs express Robo2 (Marillat et al., 2002; Kaneko et al., 2010) and type A and C cells express Slit1 (Nguyen-Ba-Charvet et al., 2004; Pennartz et al., 2004).

Slit/Robo proteins have multiple functions in non-neural and neural tissues (Blockus and Chédotal, 2016). In the CNS, they regulate midline crossing (Nguyen-Ba-Charvet et al., 1999; Plump et al., 2002; Unni et al., 2012), and are involved in SVZ-NPC migration. Namely, Slits repel SVZ-derived NPCs *in vitro* (Wu et al., 1999; Nguyen-Ba-Charvet et al., 2004; Kaneko et al., 2010), and a gradient of Slit2 in cerebral ventricles, guides NPCs along the RMS toward the OB *in vivo* (Sawamoto et al., 2006). Moreover, Slit1 is likely to facilitate the migration of SVZ-derived cells by reorganizing the RMS astrocytic tunnels (Kaneko et al., 2010, 2018). Finally, the phenotypic analysis of *Slit1* knockout mice (*Slit1*^–/–^) showed that SVZ-derived NPCs migrate more caudally and radially in Slit1-deficient animals and that *Slit1*^–/–^ neurosphere-derived cells migrate in a dispersed, rather than in a chain-like pattern (Nguyen-Ba-Charvet et al., 2004).

Although these studies suggest that Slit proteins regulate the direction and modality of migration of the SVZ cells in normal conditions and in a stroke model, their regulation and role in demyelinating conditions are still unknown. *Slit2^–/–^* mice and *Slit1/Slit2* double mutant mice die at birth. However, *Slit1*^–/–^ mice are viable and fertile. Here, we analyzed the function of Slit1 in the recruitment of SVZ-derived NPCs after CC demyelination. In parallel, we analyzed the effects of Slit1 loss on PSA-NCAM purified NPC cell migration *in vitro*. Our data show that Slit/Robo proteins are physiologically regulated in response to demyelination. Moreover, the loss of Slit1 promotes SVZ-NPCs migration *in vivo* and *in vitro* in a cell autonomous manner. These observations indicate that Slit1 plays a crucial role in regulating negatively the SVZ-NPC ectopic mobilization in response to demyelination, and could be a therapeutic target of interest to promote myelin repair by SVZ-derived progeny.

## Materials and Methods

### Animals

*Slit1* knockout mice were created by replacing a portion of an exon containing the second leucine-rich repeat domain, located in the 5′ region of the gene, with a targeting cassette containing an internal ribosome entry site (IRES), a tau-green fluorescent protein (GFP) fusion protein and a neomycin resistance gene (Plump et al., 2002). *Slit1*^+/−^ mice, which NPCs have the same migratory properties than in wild-type animals, were used as controls (Nguyen-Ba-Charvet et al., 2004). Mice were bred at the Institut de la Vision and ICM animal facilities. All experiments were performed in accordance with the European Community regulations, ICM and INSERM ethical committee (authorization 75-348; 20/04/2005).

### Lysolecithin (LPC)-Induced Demyelination

To avoid any confounding immune mediated effects, we chose the well-characterized model of LPC-induced demyelination. This model leads to acute demyelination within 2 days post-injection (dpi), SVZ-derived OPC proliferation and recruitment by the lesion peaking at 7 dpi, followed by ongoing differentiation into mature oligodendrocytes at 12 dpi and remyelination within 30 dpi (Nait-Oumesmar et al., 1999; Decker et al., 2002b). To induce demyelination, adult animals (P90) were anesthetized with 100 mg/kg body weight Ketamine (Alcyon) and 10 mg/kg body weight Xylazine (Alcyon) dissolved in 0.9% sterile saline, and positioned in a stereotaxic frame. Animals were injected unilaterally into the CC, using appropriate coordinates (1.5 mm anterior to Bregma, 1 mm lateral and 1.8 mm deep from the skull surface) with 2 μl of a 1% LPC solution (Sigma-Aldrich) in 0.9% NaCl as previously described (Caillava et al., 2011). PBS injected animals served as controls. Animals (3/4 per condition) were sacrificed 4, 6, 12 and 21 days after LPC injection.

### Real Time (RT) PCR

Adult female C57BL/6J (P90) were divided in three groups: without lesion, PBS or LPC injected (*n* = 16 of each phenotype). Seven days after PBS or LPC injection, the lateral SVZ were carefully dissected on coronal forebrain slices and dissociated. RNA was extracted using a Qiagen kit according to manufacturer recommendations (RNeasy Lipid Tissue Mini Kit Qiagen). RNA quantity and quality were evaluated using Nanodrop spectrophotometer (Thermo scientific) and a bioanalyzer, respectively (Bioanalyzer 2100, Agilent Technologies). RNA was then retro-transcribed using Invitrogen kit (Thermoscript RT PCR 11146-016) to obtain cDNA stored at −80°C. RT-qPCR was performed using Taqman method (Platinium PCR Supermix UDG, Invitrogen) according to the manufacturer instructions on ABI Prism 7000 (Applied Biosystems). Expression of *Slit1*, *Slit2*, *Slit3*, *Robo1*, and *Robo2* were quantified using corresponding primers (TaqMan Gene Expression Assays Applied Biosystems) and normalized to TBP level as a reference gene. Quantifications were conducted in triplicate for each gene and repeated in two independent experiments.

### *In vivo* SVZ Mobilization Assay

To assay ectopic mobilization of SVZ-derived progeny into the demyelinated and control corpus callosum, animals were pulse labeled with seven intraperitoneal injections of Bromodeoxy-Uridine (BrdU; 75 mg/kg body weight; Sigma-Aldrich) at 2 h intervals, the day before the stereotaxic injections of LPC/PBS as previously described (Picard-Riera et al., 2002; Tepavcevic et al., 2011).

### Tissue Processing for Immunohistochemistry

Lesioned and unlesioned animals were perfused trans-cardially with 4% PFA in cold 0.1 M phosphate buffer (PBS, pH 7.4). Brains were post-fixed in the same fixative for 1 h. Fixed tissues were then cryoprotected in 20% sucrose overnight at 4°C and frozen at −60°C in isopentane cooled in liquid Nitrogen. Twelve μm sagittal sections comprising the SVZ were cut with a cryostat (Leica, CM 3050S) and stored at −20°C for immunohistochemistry.

For immunohistochemistry, sections were incubated at room temperature for 20 min in blocking solution (PBS containing 0.2% Triton X-100 and 4% BSA). Primary antibodies were diluted using the same carrier solution and incubated overnight at 4°C. Primary antibodies were rabbit polyclonal anti-GFP (Merck Millipore) to label GFP expressing cells in *Slit1* mice, rabbit polyclonal anti-Olig2 (Merck Millipore) and anti-Sox10 (R&D System) to label the oligodendrocyte lineage, anti-platelet derived growth factor receptor alpha (PDGF-Rα, Santa Cruz Biotechnology) to label OPCs, mouse monoclonal anti-CC1 (Calbiochem) and rabbit anti-MBP (Sigma-Aldrich) to label differentiated oligodendrocytes, mouse monoclonal anti-PSA-NCAM to label NPCs (AbCys SA), mouse monoclonal anti-Ki67 (BD, Pharmingen), to label cycling cells and anti-Caspase-3 (Cell Signaling) to label cell death. Sections were washed and incubated for 1 h with species-specific secondary antibodies and counterstained with Hoechst (Sigma-Aldrich). Finally, tissue sections were washed in PBS and mounted under coverslips using Fluoromount (SouthernBiotech). Immunohistochemistry for BrdU was performed treating sections for 30 min at 37°C with HCl 2N in PBS containing 0.1% Triton X-100, then rinsed abundantly with PBS. Sections were incubated overnight at 4°C with rat monoclonal anti-BrdU antibody (AbCys SA). For double labeling, sections were first immuno-labeled with antibodies as described above, then post-fixed for 15 min in PFA 4% before BrdU pre-treatment and labeling.

### Tissue Processing for Electron Microscopy

Animals (P15 and P90, *n* = 3 of each group age and phenotype) were intra-cardially perfused with 4% PFA and 2.5% glutaraldehyde (Sigma-Aldrich) in phosphate buffer (PB, NaH_2_PO_4_, 0.2 M; Na_2_HPO_4_ 0.08 M). Brains were post-fixed in the same solution for 1 h and cut into 100 μm sections with a vibratome (Leica). Sections were then fixed in 2% osmium tetroxide (Electron Microscopy (EM) Sciences) for 30 min. After dehydration, slices were flat embedded in Epon. Ultrathin sections of the area of interest were cut using an ultra-microtome (Ultra-cut E; Reichert-Jung).

### Western Blotting

The CC of P90 brains were carefully micro-dissected. Protein extraction was performed using RIPA lysis buffer (50 mM Tris–HCl, pH 7.5, 1 mM EDTA, 1 mM EGTA, 1 mM sodium orthovanadate, 50 mM sodium fluoride, 0.1% 2-mercaptoethanol, 1% Triton X-100). A protease inhibitor cocktail (5μl/mL, Sigma-Aldrich) and the reducing agent dithiothreitol (DTT, 0.5 μl/mL, Sigma-Aldrich) were also added. Protein concentration was determined in each sample. Protein extracts were boiled for 5 min before loading onto a 10% polyacrylamide gel (Mini-Protean Precast Gels, Bio-Rad, 40 μg of protein *per* lane). Gels were then electro-transferred to a 0.2 μm PVDF membrane (Bio-Rad). Blots were blocked in 5% milk in Tris–Buffered Saline buffer (TBS, Bio-Rad) for 1 h, and then incubated at 4°C overnight with one of the following antibodies: rabbit-anti-MBP (1:500, Millipore) and rabbit-anti-α-Tubulin (1:3000, Abcam). Bands were detected with the appropriate horseradish peroxide–conjugated secondary antibodies, reacted with chemo-luminescent ECL substrate (GE Healthcare). Band intensities were measured using the ImageJ 1.37c software (National Institutes of Health, United States). Western blots were performed with the CC from *Slit1*^+/−^ and *Slit1*^–/–^ mice (*n* = 3/group). Data were averaged and presented as means ± SEM.

### Cell Culture

Cerebral hemispheres of P1 homozygous (*n* = 5) and heterozygous (*n* = 5) *Slit1*-deficient mice were dissected free of meninges and enzymatically dissociated using trypsin 0.05%. The reaction was stopped with fetal bovine serum (10%) and DNase (5 mg/ml). After centrifugation at +4°C, for 5 min, cells were collected and re-suspended in DMEM/F12 medium (1:1) supplemented with N2 supplement (1%), B27 (0,5%), insulin (25 μg/ml), glucose (6 mg/ml), Hepes (5 mM), bFGF (20 ng/ml), and EGF (20 ng/ml, Peprotech). Cells were sorted by anti-PSA-NCAM magnetic immuno-panning (Miltenyi Biotec) selection and plated on poly-ornithine/laminin. Immuno-selected PSA-NCAM + NPCs were grown for 5 days in floating conditions to generate neurospheres, or as dissociated cells for immuno-characterization or cell tracking.

### *In vitro* Cell Migration Assay

To study migration, neurospheres were plated on a poly-ornithine/laminin substrate (Sigma-Aldrich, 100 μg/ml and 10 μg/ml, respectively). NPC migration was followed by time-lapse video-microscopy (Zeiss AXIOVERT 200) for 12 h (frame every 15 min). At least 10 spheres or 30 cells were followed. Analysis of image stacks was performed using the *Metamorph* software (Ropert Scientific). Cell tracking was performed using Image-J software (National Institutes of Health, United States). Speed of migration, number of directional changes and changes in cell orientation were evaluated for each group. Changes in cell orientation were measured based on the variation of the absolute angle performed by the NPCs and defined as Δα [deg] or the angular change between the in-plane components of the most recent displacement vector (pointing from the previous point to the current point of the track) and the preceding displacement vector.

### *In vitro* Immunocharacterization

PSA-NCAM immuno-selected cells of each genotype were plated on poly-ornithine in EGF/FGF supplemented medium to assay purification efficacy. After short-term adhesion (12 h), cells were incubated at RT for 20 min in blocking solution (PBS containing 0.2% Triton X-100 and 4% BSA) fixed with PFA 4%, washed and finally incubated with primary antibodies. Mouse monoclonal anti-A2B5 (mouse IgM hybridoma from ATCC), and anti-NG2 (Chemicon) were used to identify oligodendrocyte progenitors, anti-Nestin (Merck Millipore) to identify stem/precursor cells, anti NeuN (Millipore) to identify neurons, anti-GFAP (Dako) to identify astrocytes. For A2B5 and NG2 immunostaining, cells were labeled before PFA fixation. Primary antibodies were diluted in PBS and incubated 1 h at RT. After primary antibodies, cells were rinsed and incubated for 1 h with species-specific secondary antibodies and counterstained with Hoechst (Sigma-Aldrich). Finally, cells were washed in PBS and mounted using Fluoromount (Southern Biotech).

### Imaging and Quantification

*For immunohistochemistry*, tissue sections were scanned with a Zeiss Axio Scan slide scanner microscope. Images of specific details were acquired using a Zeiss fluorescence microscope equipped with ApoTome 2. The number of Slit1-GFP^+^ cells expressing Caspase-3, or Ki67, or Olig2 combined with CC1 was quantified in defined areas of the SVZ, RMS and CC lesion at 4, 6, and 12 dpi. Quantification of Olig2 and CC1 in the non-injured brain (P15 and P90) was performed in the core of the CC. For each animal, 5,6 serial sections at 180 μm intervals were analyzed.

*For electron microscopy*, images were taken with a Philips CM 120 electron microscope. The percentage of myelinated axons over total axons and total number of axons at P15 and P90 was determined with the ImageJ software at a magnification of 62 000 for a minimum of 500 axons per animal (*n* = 2 mice/group).

### Statistics

Each n represents one animal or cell sample in the experiment. *In vivo* experiments were performed on a minimum of three mice per group. For the *in vitro* analysis, experiments were performed at least three times with NPC obtained from different dissections and dissociations. Statistical analysis was carried out using GraphPad Prism six software. All values were expressed as mean ± SEM. Normality in the variable distributions was assessed by the D’Agostino&Pearson omnibus test and Grubbs’ test was used to detect and exclude possible outliers. For Normality test, means were compared by two-tailed Student’s *t*-test. When one or both groups did not follow a normal distribution, means were compared by two-tailed Mann-Whitney *U*-test. When different independent groups were compared, we performed a one-way ANOVA plus Tukey’s multiple comparison tests. *P*-values lower than 0.05 were used as a cut-off for statistical significance.

## Results

### Slit1 Loss Does Not Alter Oligodendrocyte Numbers nor Myelination in the Postnatal and Adult Corpus Callosum

**FIGURE 1:**
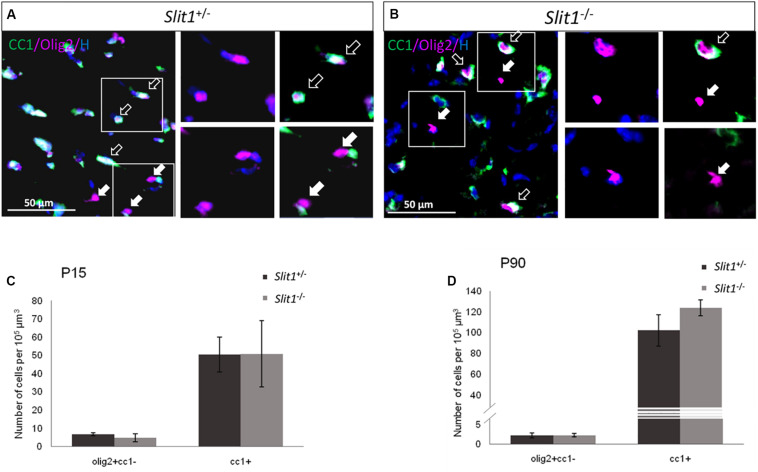
Oligodendroglial differentiation in developing and mature white matter at P15 and P90. **(A,B)** Representative illustrations of Olig2 (red) and CC1 (green) expressing cells (Hoechst, blue), in the corpus callosum of *Slit1*^+/−^ **(A)** and *Slit1^–/–^* **(B)** mice, at P90. **(C,D)**. Full arrows point to examples of Olig2^+^ OPCs and empty arrows to Olig2^+^/CC1^+^ oligodendrocytes, Quantification shows no significant difference in the number of the different cell types between *Slit1*^+/−^ and *Slit1*^–/–^ mice at P15 **(C)** or P90 **(D)**. Scale bar, 50 μm. Results are expressed as means ± SEM and analyzed with a Mann Whitney test.

**FIGURE 2:**
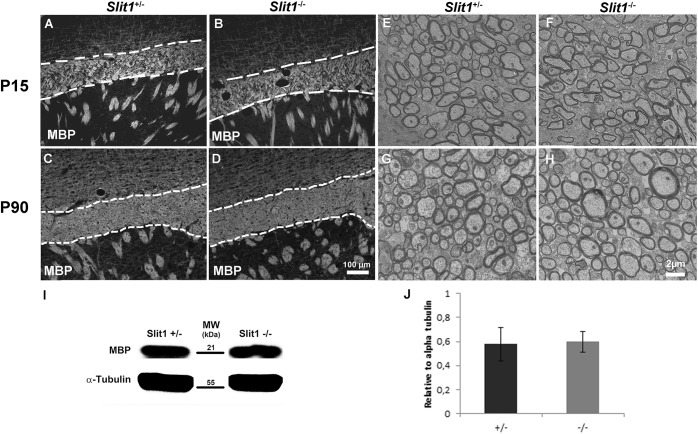
Time-course analysis of developmental myelination. **(A–D)** No difference in MBP immunodetection between *Slit1*^+/−^ **(A,C)** and *Slit1^–/–^* **(B,D)** mice on corpus callosum (broken lines) frozen section of P15 **(A,B)** and P90 **(C,D)**. **(E–H)** Electron microscopy reveals no difference in the density of myelinated axons or myelin structure between *Slit1*^+/−^ **(E,G)** and *Slit1^–/–^* **(F,H)** at P15 **(E,F)** and P90 **(G,H)**. Scale bar, 100 μm **(A–D)** and 2 μm **(E–H)**. **(I–J)** No difference in MBP levels by Western blot at P90. Results are expressed as means ± SEM and analyzed with a Student’s *t*-test, ^∗^*P* < 0.05.

As during post-natal development, CC OPCs arise mainly from the SVZ (Kessaris et al., 2006), Slit1 loss could affect the developmental process of myelination *in vivo*. To identify a possible requirement of Slit1 in the progression of CC myelination, we first quantified, in the intact CC (core) at postnatal day 15 (P15) and 90 (P90), the number of cells expressing Olig2 to identify cells of the oligodendrocyte lineage, CC1, to identify oligodendrocytes, or expressing Olig2 but not CC1, to identify OPCs. This showed no significant difference in the number of the various oligodendroglial populations between the two genotypes with, at P15: 6.8 ± 0.8 vs 5.6 ±1.2 Olig2^+^/CC1^–^ OPCs, and 50.4 ± 16.5 vs 50.8 ± 25.7 Olig2^+^/CC1^+^ oligodendrocytes, for *Slit1*^+/−^ and *Slit1*^–/–^, respectively, and at P90: 2.2 ± 0.6 and 2.2 ± 0.4 Olig2^+^/CC1^–^ OPCs, and 102.23 ± 15 and 123.70 ± 7.72 Olig2^+^/CC1^+^ oligodendrocytes for *Slit1*^+/−^ and *Slit1*^–/–^, respectively (Figure 1) suggesting that Slit1 deficiency did not alter oligodendrogenesis. Since oligodendrocytes are responsible for myelin sheath synthesis, with each cell myelinating up to 40 independent axons (Matthews and Duncan, 1971), loss of Slit1 could impact myelin synthesis. To investigate this possibility, we assessed the expression of the myelin basic protein (MBP), a major constituent of myelin (Norton and Cammer, 1984), at P15, during active myelination, and at P90, when myelination is completed. No difference in MBP immuno-reactivity was detected between *Slit1*^–/–^ mutants and controls (Figures 2A–D). We further used electron microscopy to analyze myelin fine structure and quantify the number of myelinated axons in the core of the corpus callosum, at P15 and P90. Myelin appeared normal in the absence of Slit1 (Figures 2E–H) and the percentage of myelinated axons over total axons in the CC was equivalent between heterozygous and homozygous mice (P15: *Slit1*^+/−^, 79 ± 11%; *Slit1*^–/–^, 79 ± 5%; P90: *Slit1*^+/−^, 92 ± 4%; *Slit1*^–/–^, 89 ± 0.5%) (Figures 2J,L). The absence of difference in myelination between the two phenotypes was confirmed at P90, by Western Blot quantifying MBP expression levels and using alpha-tubulin as control (*Slit1*^+/−^, 0.58 ± 0.14; *Slit1*^–/–^, 0.59 ± 0.08; *P* = 0.82) (Figure 2I). As Slits also regulate the development of CC axons (Unni et al., 2012), we also examined whether the total number of axons at P90 was modified, but found no difference between *Slit1*^+/−^ and *Slit1*^–/–^ mice (*Slit1*^+/−^, 911.027 ± 32.12 axons/mm^2^; *Slit1*^–/–^, 882.551 ± 15.25 axons/mm^2^; *P* = 0.16; results were analyzed with a *t*-test).

### Transcriptional Regulation of Slits and Robos After LPC-Induced Demyelination of the Corpus Callosum

**FIGURE 3:**
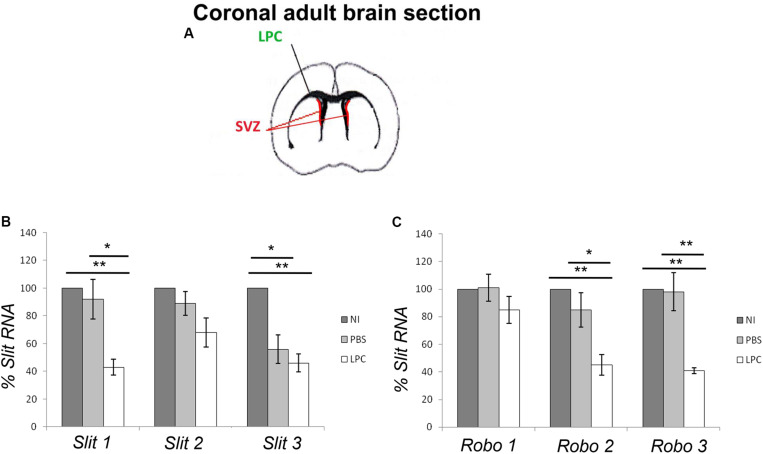
Slit and Robo real-time PCR quantification. **(A)** Representative scheme of a coronal section of the adult brain highlighting the SVZ area selected for the study. **(B)** *Slit1* and *Slit3* expression levels are significantly repressed in SVZ cells from LPC injected animals. **(C)** Slits down regulation is correlated with a significant decrease in *Robo2 and Robo3* expression levels. NI, not injected, LPC, lysolecithin, SVZ, subventricular zone. Results are expressed as means ± SEM, analyzed with one-way ANOVA plus Tukey’s multiple comparison tests ^∗^*P* < 0.05, ^∗∗^*P* < 0.01 and are reported to the percentage of non-injected controls.

Previous reports indicated that transcripts/proteins for *Slits* and their receptors *Robos*, are highly expressed in the periventricular and ventricular areas of the adult brain in non-pathological conditions with *Slit1* and *Robo2* and *Robo3* expressed in the adult SVZ/RMS (Nguyen-Ba-Charvet et al., 2004; Kaneko et al., 2018). On the other hand, LPC-induced demyelination activates the SVZ and enhances ectopic migration of SVZ-derived NPCs to the lesion with maximal recruitment by 7 days (Nait-Oumesmar et al., 1999; Decker et al., 2002b). Since the absence of Slit1 induces ectopic migration of SVZ-NPCs in unlesioned animals, we hypothesized that Slit1 could be physiologically regulated in response to LPC-induced demyelination. To investigate this possibility, we first analyzed the expression of Slits and Robos transcripts in the adult SVZ in response to LPC induced demyelination of the corpus callosum at 7 dpi. To this end, the lateral SVZ from non-injected, PBS or LPC injected wild-type mice were carefully micro-dissected from coronal brain sections for RNA extraction (Figure 3A). RT-PCR showed that the levels of expression of *Slit1* and *Slit3* transcripts, but not *Slit2*, were significantly repressed in SVZ cells harvested from LPC injected animals compared to non-injected, and PBS injected animals (Figure 3B). This was correlated with a significant decrease in the expression of *Robo2 and Robo3* transcripts, *Robo2* being the preferential partner of *Slit1* in the SVZ (Figure 3C).

### Slit1 Loss Enhances Ectopic Dispersal and Recruitment of SVZ-NPCs Into the Demyelinated Corpus Callosum

**FIGURE 4:**
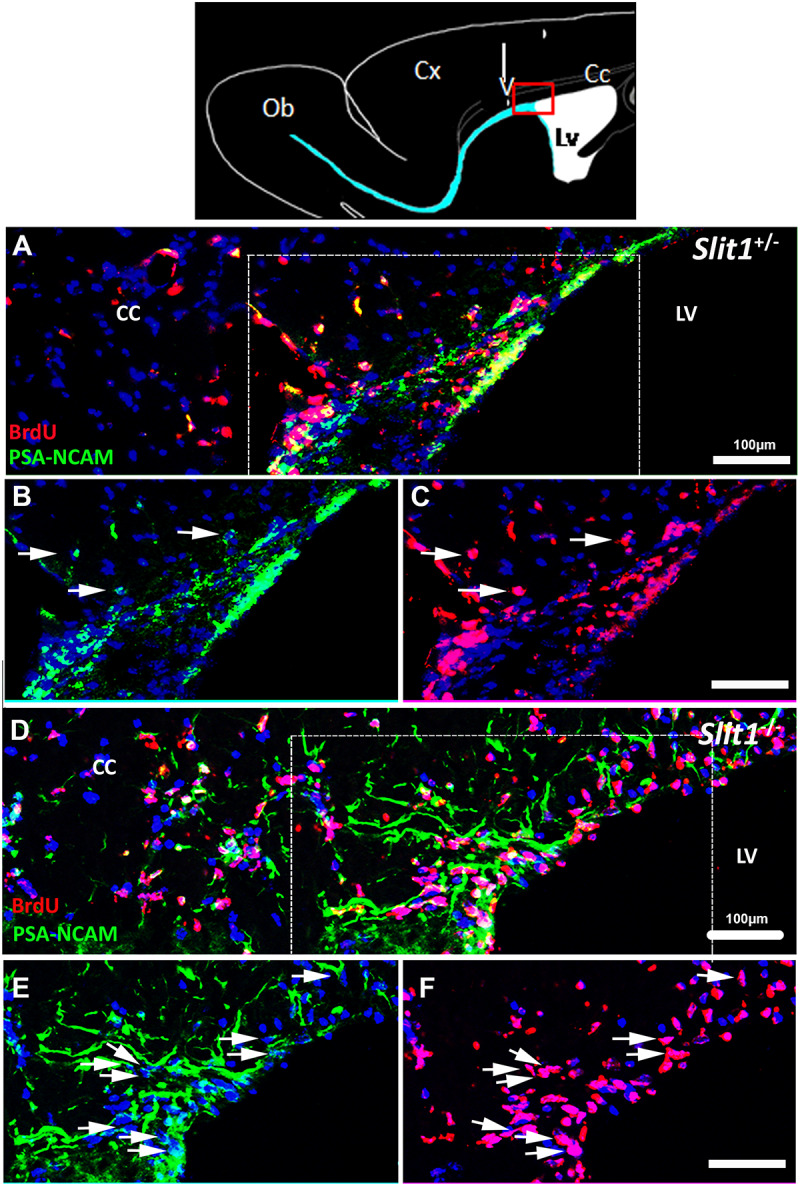
Dispersal of SVZ-derived progenitors into the corpus callosum in response to LPC- induced demyelination of the CC. The top scheme represents a forebrain sagittal section with the area of interest (red box), and the area of LPC injection (arrow). **(A–F)** The lesion is out of the field, and localized in the upper left corner of the images. The SVZ-derived progeny was identified by double labeling for BrdU and the NPC marker, PSA-NCAM at 6 dpi. BrdU^+^/PSA-NCAM^+^ cells are more dispersed in the demyelinated corpus callosum in *Slit1^–/–^* **(D–F)** compared to *Slit1*^+/−^ **(A–C)** mice. **(B,C)** and **(E,F)** are single channels and enlarged views of A and D, respectively, illustrating that the majority of BrdU traced cells (red) express PSA-NCAM (green). Scale bar, 100 μm. LV, lateral ventricle; CC, Corpus Callosum.

Previous observations indicated that in *Slit1*^–/–^ mice, GFP is expressed by type A (neuroblasts) and type C cells (intermediate progenitors) and that the loss of Slit1 in physiological conditions, promotes SVZ and RMS derived cells into the periventricular structures and especially in the CC (Nguyen-Ba-Charvet et al., 2004, and above results). Mobilized GFP^+^ cells are known to express PSA-NCAM (Nguyen-Ba-Charvet et al., 2004), and GFP expression is down-regulated in SVZ-derived progeny when maturing in glial cells in the corpus callosum, or neuronal cells in the OB (Nguyen-Ba-Charvet et al., 2004; Kaneko and Sawamoto, 2009). To investigate the consequence of *Slit1* deletion on SVZ-progeny ectopic mobilization and recruitment in response to demyelination, and avoid only partial tracking of SVZ-derived progeny due to GFP down-regulation P90 *Slit1*^–/–^ and *Slit1*^+/−^ mice were injected intra-peritoneally with BrdU 1 day before the LPC injection and their brain collected 4, 6, 12 dpi. This method of LPC-induced demyelination of the CC is commonly used to assess SVZ-derived progeny recruitment in lesion sites (Nait-Oumesmar et al., 1999; Decker et al., 2002a, b; Picard-Riera et al., 2004). Immuno-detection of BrdU on sagittal sections at 6 dpi, confirmed the more dispersed migration of PSA-NCAM^+^/BrdU^+^ cells in *Slit1*^–/–^ animals compared to controls (Figure 4). In both genotypes, the majority of GFP^+^ cells expressed BrdU but not all BrdU^+^ cells expressed GFP, supporting the down-regulation of GFP with maturation of mobilized cells.

**FIGURE 5:**
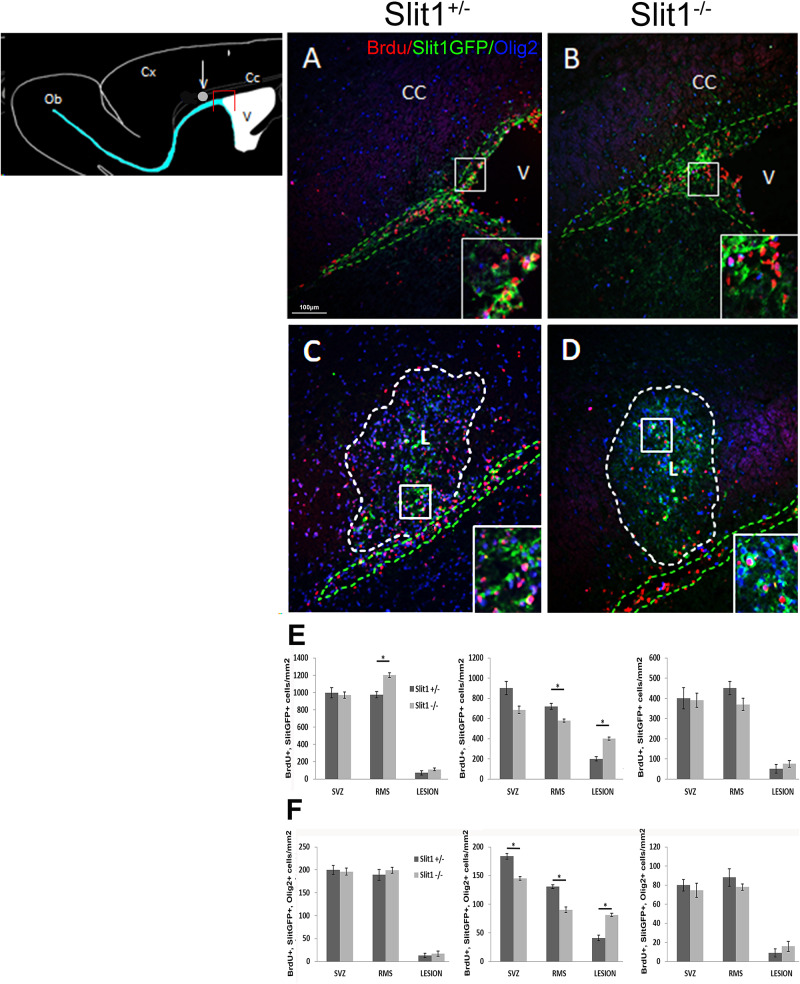
Recruitment of the SVZ-derived progeny by the LPC-induced lesion of the corpus callosum at 6 dpi. The scheme represents the areas of interest: the lesion (white circle) and the SVZ (red box). **(A–D)** illustrates the presence of BrdU^+^ and Slit-GFP^+^ cells on sagittal forebrain sections at the levels of the **(A,B)** SVZ and **(C,D)** corpus callosum lesion of *Slit1*^+/−^ **(A,C)** and *Slit1*^–/–^ **(C,D)** mice. Insets are higher magnifications illustrate Slit-GFP^+^ cells expressing BrdU. **(E,F)** Quantification at the level of the SVZ, RMS and lesion at four (left graphs), six (middle graphs) and 12 dpi (right graphs) indicates that BrdU^+^/Slit1-GFP^+^ **(E)** and BrdU^+^/Slit-GFP^+^/Olig2^+^ **(F)** cells colonize the lesion more efficiently in *Slit1*^–/–^ animals, than in *Slit1*^+/−^ controls. Note that parenchymal Olig2^+^ cells including adult OPC, do not express GFP. Insets illustrate BrdU^+^/GFP^+^ cells at higher magnifications. Broken lines in green delineate the SVZ **(A,B)** and RMS **(C,D)** and in white, define the lesion **(C,D)**. V, lateral ventricle; CC, Corpus Callosum, L, lesion. Scale bar, 100 μm. Results are expressed as means ± SEM and analyzed with a Student’s *t*-test, ^∗^ = *P* < 0.05.

To further understand the role of Slit1 in SVZ-NPC recruitment by the lesion, we next evaluated the number of GFP^+^/BrdU^+^ cells in the SVZ, RMS (Figures 5A,B,E) and the lesion (**Figures 5C,D,E**) at 4, 6 and 12 dpi into the CC. The recruitment of GFP^+^/BrdU^+^ cells was maximal at 6 dpi, as previously described (Nait-Oumesmar et al., 1999; Caillava et al., 2011) with a two-fold increase of GFP^+^/BrdU^+^ cells in the lesions of *Slit1*^–/–^ mice (399 ± 16 cells/mm^2^) compared to *Slit1*^+/−^ mice (199 ± 21 cells/mm^2^) and a corresponding differential depletion in the SVZ and RMS of *Slit1*^–/–^ mice (SVZ: 685 ± 38 and RMS: 580 ± 15) compared to *Slit1*^+/−^ mice (SVZ: 900 ± 66 and RMS: 720 ± 29) (Figure 5E, *P* < 0.05, *t*-test). The enhancement of the SVZ-NPC recruitment into the lesion was specific of demyelinating conditions as recruitment of lower amplitude was observed in the corpus callosum of PBS injected (*Slit1*^+/−^, 0.5 ± 0.002 cells/mm^2^, *Slit1*^–/–^, 0.7 ± 0.001 cells/mm^2^, *P* = 0.22, *t*-test) and non-injected mice (Nguyen-Ba-Charvet et al., 2004). Because SVZ-derived cells can be recruited by the LPC-induced focal demyelination and differentiate into cells of the oligodendrocyte lineage, we investigated the impact of Slit1 deficiency on these events. Triple detection of BrdU, GFP and Olig2, indicated that parenchymal Olig2^+^ cells of the CC did not express GFP. Moreover, oligodendroglial cell recruitment was enhanced significantly by two-folds at 6 dpi in response to demyelination in the absence of Slit1 (81.2 ± 2.9 cells/mm^2^) compared to *Slit1*^+/−^ mice (41 ± 4.9 cells/mm^2^; *P* < 0.05, *t*-test; Figure 5F). These findings further imply that Slit1 is an important negative modulator of ectopic migration and recruitment by the lesion of SVZ-derived progeny including those committed toward the oligodendrocyte lineage.

### Slit1 Loss Does Not Affect NPC Proliferation, nor Cell Death in the Adult Corpus Callosum After LPC-Induced Demyelination

In the LPC model, demyelination is completed within 2 days after toxin injection, and is followed by increased cell proliferation in both the adult SVZ/RMS and the lesion during the first week after LPC injection (Nait-Oumesmar et al., 1999; Caillava et al., 2011). Although the sole absence of Slit1 does not affect proliferation in the adult SVZ, in normal conditions (Nguyen-Ba-Charvet et al., 2004; Borrell et al., 2012), we investigated the possible role of Slit1 in the proliferation in the adult anterior SVZ, and RMS of adult *Slit1*^+/−^ and *Slit1*^–/–^ mice, in response to demyelination. To this end, we quantified the number of GFP/Ki67 double-positive cells at 4, 6 and 12 dpi (Supplementary Figure S1). As expected, proliferation in the SVZ/RMS was maximal at 4 dpi with a two-fold reduction of the total number of GFP^+^/Ki67^+^ cells proliferating in the SVZ, RMS and lesion, at 6 dpi and five to six-folds reduction at 12 dpi compared to 4 dpi of both groups. However, there was no statistical difference between *Slit1*^+/−^ and *Slit1*^–/–^ mice for each location, at all time points analyzed (Supplementary Figures S2M–O). These results suggest that adult SVZ-NPC proliferation after CC demyelination is either Slit1 independent or efficiently compensated by other Slits.

Since the increase in ectopic migration, observed at 6 dpi, in *Slit1*^–/–^ brains could also have resulted from altered cell survival in response to demyelination, we assessed the potential contribution of Slit1 to apoptosis. Caspase-3^+^ cells were quantified in the SVZ, RMS, and lesion at 6 dpi of LPC (Supplementary Figure S2). The number GFP^+^/Caspase-3^+^ cells was very low and not different in both groups (<0.01% of cells).

These observations rule out a possible contribution of altered proliferation or survival in the enhanced recruitment of SVZ-derived NPCs by the lesion resulting from Slit1 loss.

### Slit1 Function in NPC Migration Is Cell Autonomous

**FIGURE 6:**
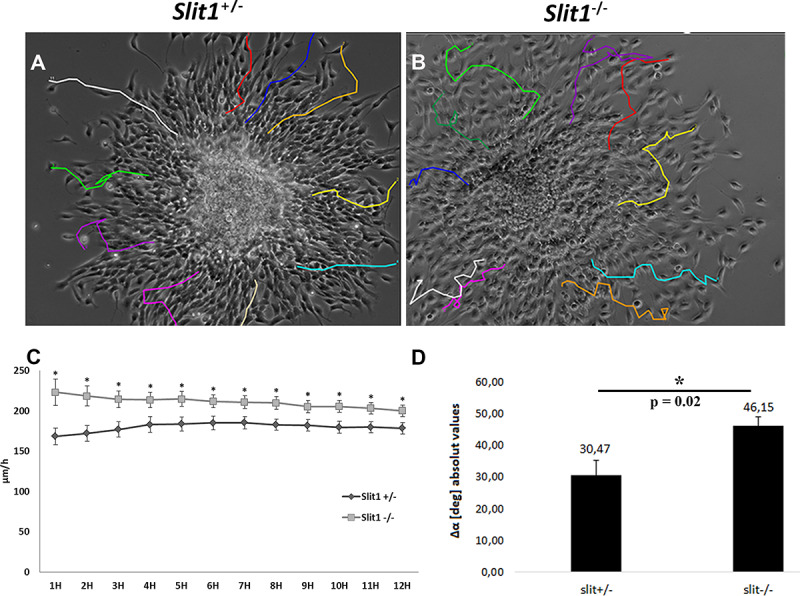
*Slit1^–/–^* Migration modalities of NPCs by time-lapse video recording. PSA-NCAM immuno-selected precursors were plated as neurospheres **(A,B,D)** or as dissociated cells **(C)** on Poly-ornithine/laminin. Cell migration was followed by time-lapse video microscopy during a period of 12 h. **(C)** NPCs from *Slit1^–/–^* animals showed a higher speed of migration than controls. **(D)** NPCs escaping from neurospheres show significant changes in amplitude direction. Results are expressed as means ± SEM and analyzed with a Student’s *t*-test, **(C)**^∗^*P* < 0.05, **(D)**^∗^*P* = 0,02.

It has been suggested that the role of Slit1 in SVZ/RMS cell migration is primarily non-autonomous (Kaneko et al., 2010). We previously demonstrated *in vitro* that neurospheres obtained from *Slit1*^–/–^ newborn mice migrate abnormally, when seeded on poly-ornithine/collagen, for 5 days, in medium containing or not, EGF and FGF-2 (Nguyen-Ba-Charvet et al., 2004). However, neurospheres are a mixture of stem/progenitor cells, which render the data interpretation complex with respect to cell autonomy. Since Slit1 expression is prominent in PSA-NCAM^+^ progenitors (Pennartz et al., 2004), and in order to use an homogeneous cell population, we re-addressed the function of Slit1 in NPC migration using purified preparations of immuno-selected PSA-NCAM^+^ NPCs. We first examined the antigenic profile of the purified PSA-NCAM^+^ NPCs after short-term culture, using the NPC markers, Nestin, and PSA-NCAM, the combined OPC markers NG2/A2B5, the astrocyte marker GFAP, and neuronal marker NeuN. The majority of *Slit1*^+/−^ and *Slit1*^–/–^ PSA-NCAM sorted NPCs (identified by Hoechst staining) expressed the Nestin (91 ± 1.6 vs 90 ± 0.5%) and PSA-NCAM (94 ± 5.5 vs 90 ± 2.1%), with few cells expressing NG2/A2B5, and none expressing GFAP and NeuN. Migration was followed by time-lapse video-microscopy plating either PSA-NCAM^+^ spheres or dissociated cells on a poly-ornithine/laminin substrate. *In vivo* analysis of sphere-derived cells indicated a greater dispersion of NPCs in the SVZ/RMS of *Slit1*^–/–^ compared to controls suggesting that they were miss-oriented. To gain insights into the pattern of migration, we analyzed the number and angular amplitudes of directional changes performed by the NPCs. Spheres were used to induce radial chain-like migration (Figures 6A,B). Quantification of migration speed of dissociated PSA-NCAM^+^ NPCs showed that *Slit1*^–/–^ NPCs, migrated significantly faster than *Slit1*^+/−^ cells at all times tested (Figure 6C). NPCs derived from *Slit1*^–/–^ spheres changed their direction more frequently (6.5 ± 1.5 times per cell) for *Slit1*^+/−^, vs 8 ± 2.0 for *Slit^–/–^*; *P* = 0.02, *t*-test) and had significantly larger angular amplitudes (Δα deg of *Slit1*^+/−^, 30.47 ± 4.93; *Slit1*^–/–^ 46.35 ± 2.98, *P* = 0.01, *t*-test) compared to *Slit1*^+/−^-derived NPCs (Movie, Figure 6D). These data indicate that Slit1 function in NPC migration is cell autonomous.

## Discussion

Remyelination failure may result from defects in proliferation, migration and/or differentiation of cells of the oligodendroglial lineage. Therefore the identification of extrinsic and intrinsic factors governing progenitor cell proliferation, migration and differentiation into oligodendrocytes is crucial to elucidate the mechanisms of postnatal neurogenesis and gliogenesis. Because adult SVZ-OPCs and NPCs contribute to myelin repair in the mammalian forebrain (Nait-Oumesmar et al., 1999; Picard-Riera et al., 2002; Menn et al., 2006; Aguirre et al., 2007; Jablonska et al., 2010; Xing et al., 2014; Brousse et al., 2015), identifying these factors is also essential to understand spontaneous remyelination, which in diseases such as MS is insufficient to ensure clinical recovery. Although our knowledge of signals that regulate SVZ-derived OPC and NPC cell proliferation and differentiation is quite extensive, those that regulate SVZ cell migration/recruitment are less well understood. More specifically, the role of Slit proteins in SVZ ectopic cell migration in response to demyelination has not been addressed.

Here, we used *Slit1* knockout mice to investigate the functional role of this axon guidance molecule in NPC/OPC migration in myelination and in response to demyelination. Combining *in vitro* and *in vivo* experiments, we provide evidence that Slit1 loss alters adult NPC/OPC migration *in vitro* and *in vivo* under pathological conditions without affecting NPC cell proliferation, survival or developmental myelination.

Corpus callosum OPCs arise mainly from the SVZ during post-natal development (Kessaris et al., 2006). We first show that Slit1 loss has no effect on oligodendrocyte differentiation during early postnatal development since the relative proportions of OPC/mature oligodendrocytes were identical in control and *Slit1*^–/–^ mice. This is further supported by the normal levels of the MBP protein, number of myelinated axons and g ratios in *Slit1*^–/–^ mice. This absence of developmental myelination defects in *Slit1*^–/–^ mice might result from compensatory mechanisms as other Slits are expressed during forebrain development (Marillat et al., 2002; Plump et al., 2002).

Slits/Robos proteins can act as repellents and are expressed by NPCs. We hypothesized that the expression of these proteins could act as a brake in type A and type C cells slowing down their exit from the SVZ/RMS. Our previous work showed that in physiological conditions Slit1 disruption disorient SVZ precursors from their olfactory bulb destiny (Nguyen-Ba-Charvet et al., 2004). The present findings strengthen the hypothesis that disruption of Slit1 function has a positive impact on NPC ectopic migration into the corpus callosum, leading to enhanced cell recruitment by the lesion. This effect was specific, as the lack of Slit1 did not affect cell proliferation or survival. Moreover, it was specific of SVZ-derived progeny as Slit-GFP is not express by parenchymal OPCs (see Figure 5). This is in contrast with molecules such as Anosmin1, which are expressed by SVZ-NPCs and OPCs, and, which modulation affects the development of both populations including oligodendrocyte differentiation and myelination (Murcia-Belmonte et al., 2016). The role of Slit1 in preventing NPC migration from the SVZ in normal conditions was further supported by the fact that under pathophysiological conditions, *Slits* and *Robos* are down-regulated in the SVZ at the transcriptional level, 7 days post demyelination, and thus concomitantly with NPC recruitment at the lesion. Of particular interest was the specific down regulation of *Slit1* and *Robo2*, which act as partners within the SVZ/RMS system (Kaneko et al., 2018). These observations are consistent with the idea that Slit1 in normal conditions prevents SVZ cell mobilization to ectopic locations and that, consequently, they are down regulated to allow NPC ectopic dispersal from the SVZ in response to demyelination.

In the absence of Slit1, SVZ-derived NPC migrate in a more dispersed mode toward the lesion than control NPCs suggesting that Slit1 plays a role in NPC directed migration. This was confirmed by the time-lapse analysis of MACS-sorted PSA-NCAM^+^ NPC cell migration as changes in both cell orientation and direction were observed in the absence of Slit1. As these purified NPC cultures were devoid of astrocytes or any other cell types, our data imply that directed migration/orientation of NPCs acts through a cell-autonomous mechanism involving possibly the interaction between Slit1 and Robos expressed by sister cells (Nguyen-Ba-Charvet et al., 2004). Our results thus challenge those provided by Kaneko and colleagues, suggesting that NPC migration in the adult brain acts via a non-autonomous mechanism resulting solely from the interaction of neuroblasts with astrocytes expressing Robo and Slit, respectively (Kaneko et al., 2010). Our *in vitro* data provide additional support for a role for Slits as intrinsic regulators of NPC directed migration, without excluding an additional role as an extrinsic regulator of NPC migration across astrocytes. Recent data indicate that Slit-Robo signaling, mediates rapid and dynamic changes in the actin cytoskeleton of reactive astrocytes to maintain the route for neuronal migration toward a lesion induced by stroke (Kaneko et al., 2018). While astrocyte reactivity is also triggered by LPC-induced demyelination, lesions are acute, and far less severe than stroke, inducing a minor scar after NPC/OPC recruitment, suggesting a minor impact of the astrocyte Slit-Robo regulation during NPC recruitment by the LPC-lesion.

Several studies unraveled the presence of cellular and molecular cues regulating ectopic migration/recruitment of SVZ-derived NPC in pathological conditions (Spigoni et al., 2014). Such cues are mainly present in the environment (extrinsic cues) and include sequentially, disruption of the astrocyte furrow liberating neural progenitors from their physical constrain (Nait-Oumesmar et al., 1999), lesion-induced vascular remodeling via Netrin1, guiding and promoting progenitor emigration to the demyelinating site (Cayre et al., 2013), and glial-derived neurotrophins such as CNTF (Vernerey et al., 2013) and chemokines such as SDF1 via CXCR4 (Imitola et al., 2004) attracting the emigrating progenitors to the lesion. Only few reports indicate the role of intrinsic cues. Polysialylated residues (PSA) on NCAM by facilitating homophilic NPC interactions (Hu et al., 1996), enhances NPC migration to the olfactory bulb and prevents their efficient recruitment by the lesion (Decker et al., 2002a). In physiological conditions, Slit1 orients NPCs toward the OB, refraining (negative regulator) their dispersion away from the SVZ/RMS niche. Here we show, that their genetically programmed down-regulation in demyelinating conditions in the presence of PSA-NCAM expression, is sufficient to disorient NPCs and contribute with vascular remodeling and lesion-derived chemo-attractants, to their efficient recruitment by the lesion.

Very little is known about the molecular mechanisms down-regulating Slit and Robo expression in response to demyelination. These may include unknown factors of the environment and/or the SVZ-progeny maturation status, decreasing Slit1 expression with cell differentiation (Kaneko et al., 2018). One possibility could be that the lesion changes the local expression of molecules such as FGF and Wnts both of which influence oligodendrocyte production in the SVZ (Chavali et al., 2018; Kang et al., 2019) and can modulate the expression of *Robo* and *Slit* genes (Borrell et al., 2012). The lesion might also perturb neuronal activity locally and it is known that for example, during development, neuronal activity can also modulate Robo expression (Mire et al., 2012).

Slit proteins could play redundant roles. Our analysis by RT-PCR shows that all *Slit* and *Robo* transcripts were down regulated in response to corpus callosum demyelination. *Slit2*, which is expressed by periventricular tissue (septum and choroid plexus) was not significantly down regulated in response to LPC. *Slit3* was significantly repressed. However, it is minimally expressed by SVZ precursors and does not bind the major SVZ-NPC *Robo2* partner. These observations suggest that *Slit2* or *Slit3* are unlikely to act as redundant partners in the regulation of SVZ-ectopic migration in response to LPC. Despite the potential redundant function of the Slit/Robo family during forebrain development, *Slit1* and *Robo2* transcripts, which proteins are exclusively expressed by the adult SVZ (Nguyen-Ba-Charvet et al., 2004; Kaneko et al., 2010, 2018) were significantly down-regulated. Therefore, the present study highlights for the first time a crucial role for Slit1 as a regulator of adult NPC ectopic migration in response to demyelination. Although future studies should investigate the regulation of Slits/Robos in a more relevant animal model of MS and MS tissue, the present findings could contribute to a better understanding of remyelination failure in MS and be crucial to design novel therapeutic approaches targeting Slit/Robos to enhance the SVZ contribution to CNS remyelination in demyelinating diseases.

## Data Availability Statement

The Western blot data used to support the findings of this study are available from the corresponding author upon request.

## Ethics Statement

Experiments were performed according to European Community regulations and INSERM Ethical Committee (authorization 75-348; 20/04/2005) and were approved by the local Darwin Ethical Committee.

## Author Contributions

CC, GS, BG-D, CB, and AB-V contributed conception and design of the study. GS and CC organized the database. CD, GS, BG-D, and NS performed the statistical analysis. AY expanded and provided transgenic animals, AB-V wrote the first draft of the manuscript. CC, GS, BG-D, NS, AY, and AC wrote sections of the manuscript. All authors contributed to manuscript revision, read and approved the submitted version.

## Conflict of Interest

The authors declare that the research was conducted in the absence of any commercial or financial relationships that could be construed as a potential conflict of interest.

## Supplementary Material

FIGURE S1 Proliferation of Slit1-GFP cells in response to demyelination. The orientation of SVZ and lesion is similar to the scheme of Figure five but tilted at 90° to the right. **(A–L)** Representation of double labeled Ki67, Slit1GFP at 6 dpi **(A–F)** in the SVZ, and **(G–L)** in the lesion. Insets are enlargements of Ki67/GFP double-labeled cells in C, F, I, and L, respectively. **(M–O)** Quantification of double-labeled Ki67/Slit1GFP cells indicates an increase of proliferation in the SVZ and the lesion at 4 dpi compared to 6 and 12 dpi but no differences in proliferation between *Slit1*^+/−^ and *Slit1*^–/–^ mice. Broken lines delineate the SVZ in **(A–F)**, and the lesion in **(G–L)**. V, lateral ventricle; CC, Corpus Callosum; L, lesion. Scale bar 50 μm. Results are expressed as means ± SEM and analyzed with a Student’s *t*-test.

FIGURE S2 NPC cell death in response to demyelination in the adult brain. The orientation of SVZ and lesion is similar to the scheme of Figure 5 but tilted at 90° to the right. **(A–D”)** At 6 dpi, apoptotic cells identified by Caspase three immuno-labeling, were not found in the adult SVZ/RMS **(A–B”)** nor in the demyelinated CC **(C–D”)** of *Slit1*^+/−^ and *Slit1*^–/–^ mice. **(E–F”)**, enlarged view of Caspase3^+^ cells in the olfactory bulb of the same mice. Broken lines delineate the SVZ **(A–B”)**, and the lesion **(C–D”)**. LV, lateral ventricle; CC, Corpus Callosum; L, Lesion. Scale bar 100 μm **(A–D”)** and 10 μm **(E–F”)**.
