# Supplementary figures and images for: Slit1 Protein Regulates SVZ-Derived Precursor Mobilization in the Adult Demyelinated CNS
Source: Front Cell Neurosci. 2020 Jun 26;14:168. doi: 10.3389/fncel.2020.00168 (PMC7332780; doi:10.3389/fncel.2020.00168)

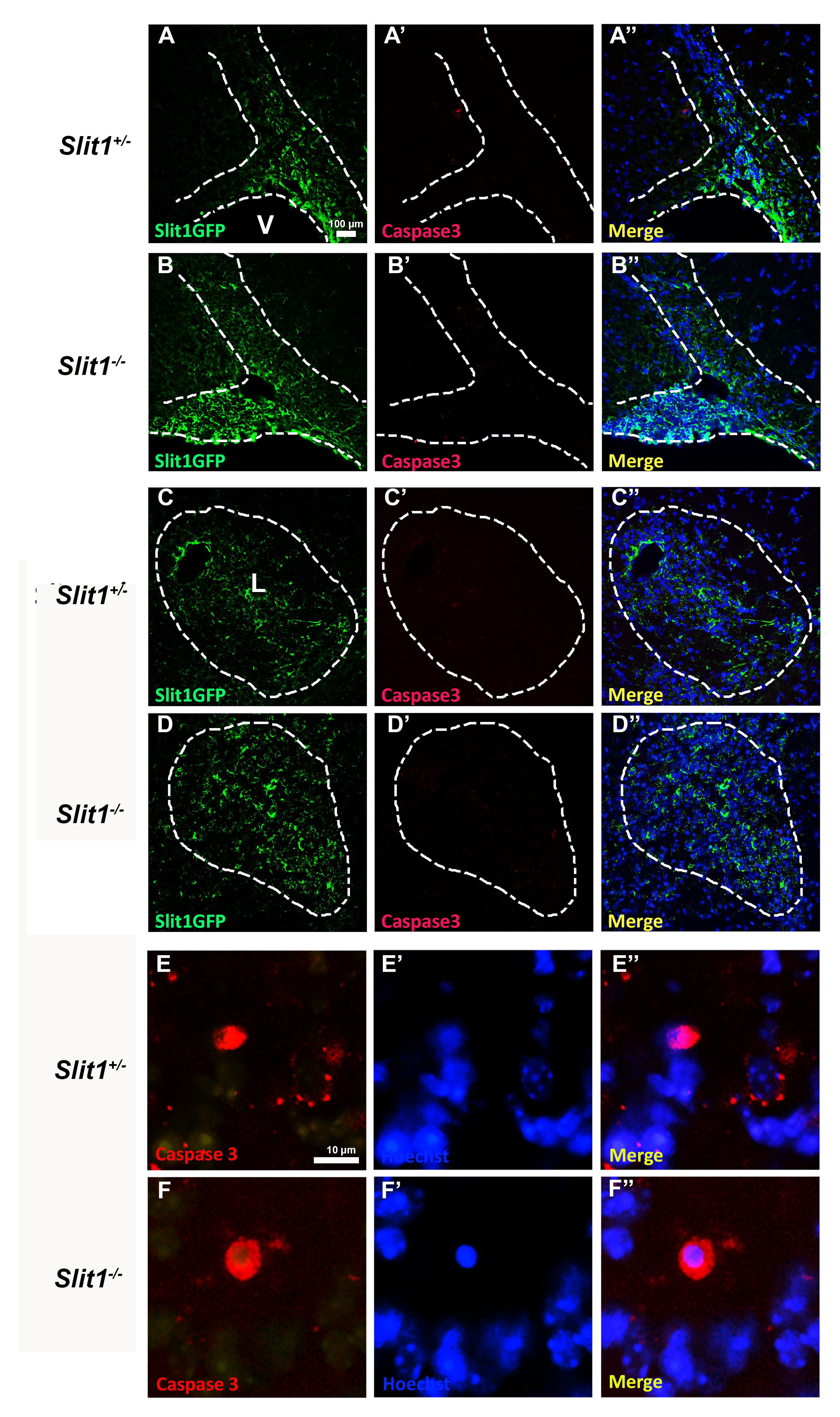

Supplement: FIGURE S1 — Proliferation of Slit1-GFP cells in response to demyelination. The orientation of SVZ and lesion is similar to the scheme of Figure five but tilted at 90° to the right. (A–L) Representation of double labeled Ki67, Slit1GFP at 6 dpi (A–F) in the SVZ, and (G–L) in the lesion. Insets are enlargements of Ki67/GFP double-labeled cells in C, F, I, and L, respectively. (M–O) Quantification of double-labeled Ki67/Slit1GFP cells indicates an increase of proliferation in the SVZ and the lesion at 4 dpi compared to 6 and 12 dpi but no differences in proliferation between Slit1+/− and Slit1–/– mice. Broken lines delineate the SVZ in (A–F), and the lesion in (G–L). V, lateral ventricle; CC, Corpus Callosum; L, lesion. Scale bar 50 μm. Results are expressed as means ± SEM and analyzed with a Student’s t-test. [file Image_1.tif]

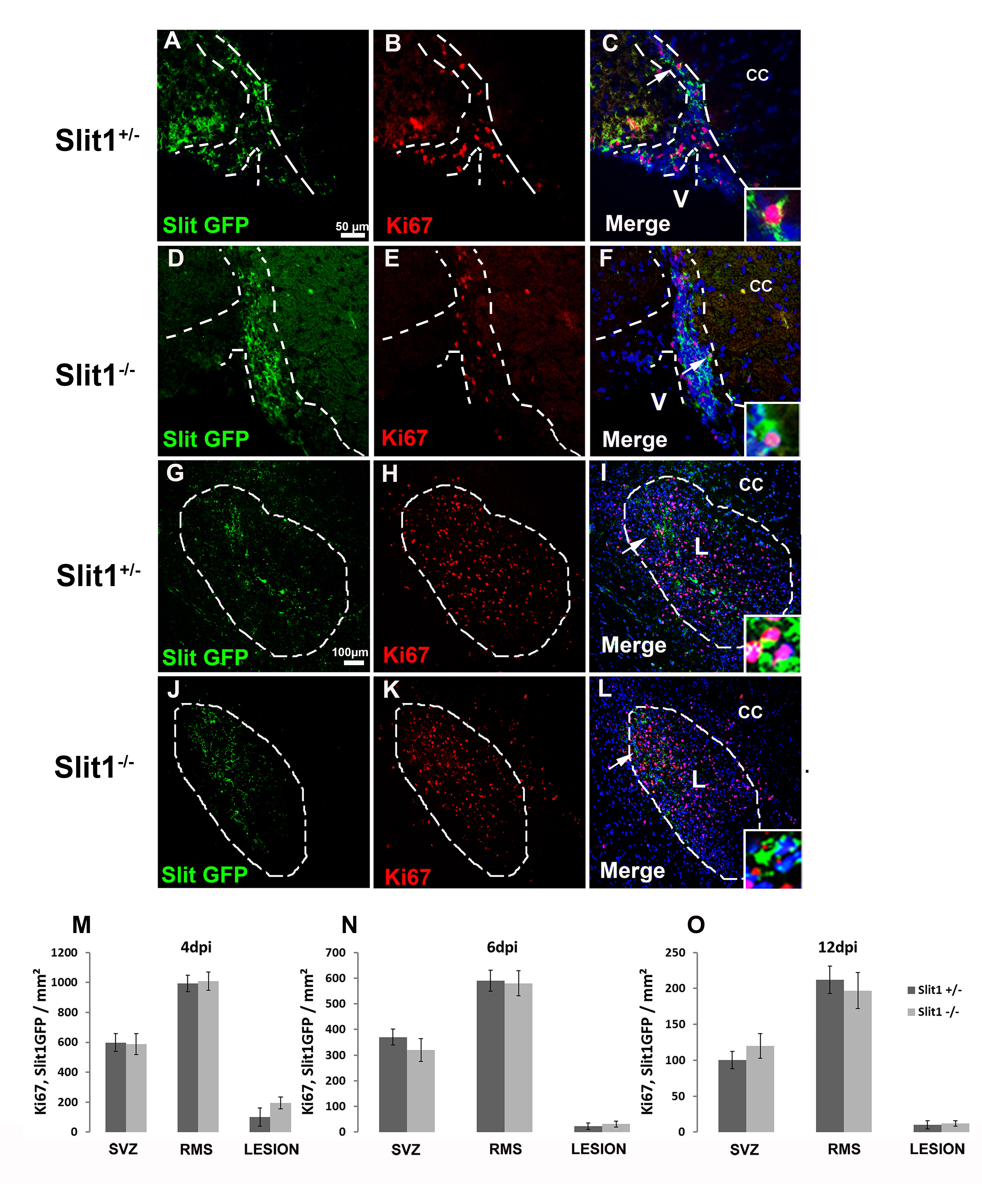

Supplement: FIGURE S2 — NPC cell death in response to demyelination in the adult brain. The orientation of SVZ and lesion is similar to the scheme of Figure 5 but tilted at 90° to the right. (A–D”) At 6 dpi, apoptotic cells identified by Caspase three immuno-labeling, were not found in the adult SVZ/RMS (A–B”) nor in the demyelinated CC (C–D”) of Slit1+/− and Slit1–/– mice. (E–F”), enlarged view of Caspase3+ cells in the olfactory bulb of the same mice. Broken lines delineate the SVZ (A–B”), and the lesion (C–D”). LV, lateral ventricle; CC, Corpus Callosum; L, Lesion. Scale bar 100 μm (A–D”) and 10 μm (E–F”). [file Image_2.jpeg]
